# Supplementary material for: Smartphone-delivered mental health care interventions for refugees: A systematic review of the literature
Source: Glob Ment Health (Camb). 2022 Dec 23;10:e6. doi: 10.1017/gmh.2022.61 (PMC9947632; doi:10.1017/gmh.2022.61)
Supplement: Supplementary file 1 [file S2054425122000619sup001.docx]

**Online Supplement 1**

*Preferred Reporting Items for Systematic Reviews and Meta-Analyses Guidelines* (PRISMA; Liberati et al., 2009)

| **Section and Topic** | **Item #** | **Checklist item** | **Location where item is reported** |
| --- | --- | --- | --- |
| **TITLE** | | |  |
| Title | 1 | Identify the report as a systematic review. | p. 1 |
| **ABSTRACT** | | |  |
| Abstract | 2 | See the PRISMA 2020 for Abstracts checklist. | Partly fulfilled |
| **INTRODUCTION** | | |  |
| Rationale | 3 | Describe the rationale for the review in the context of existing knowledge. | p. 4 – 5 |
| Objectives | 4 | Provide an explicit statement of the objective(s) or question(s) the review addresses. | p. 5 |
| **METHODS** | | |  |
| Eligibility criteria | 5 | Specify the inclusion and exclusion criteria for the review and how studies were grouped for the syntheses. | p. 6 |
| Information sources | 6 | Specify all databases, registers, websites, organisations, reference lists and other sources searched or consulted to identify studies. Specify the date when each source was last searched or consulted. | p. 6 – 7 |
| Search strategy | 7 | Present the full search strategies for all databases, registers and websites, including any filters and limits used. | p. 6 – 7 |
| Selection process | 8 | Specify the methods used to decide whether a study met the inclusion criteria of the review, including how many reviewers screened each record and each report retrieved, whether they worked independently, and if applicable, details of automation tools used in the process. | p. 6 – 8 |
| Data collection process | 9 | Specify the methods used to collect data from reports, including how many reviewers collected data from each report, whether they worked independently, any processes for obtaining or confirming data from study investigators, and if applicable, details of automation tools used in the process. | p. 8 – 9 |
| Data items | 10a | List and define all outcomes for which data were sought. Specify whether all results that were compatible with each outcome domain in each study were sought (e.g., for all measures, time points, analyses), and if not, the methods used to decide which results to collect. | p. 8 – 9 |
|  | 10b | List and define all other variables for which data were sought (e.g., participant and intervention characteristics, funding sources). Describe any assumptions made about any missing or unclear information. | p. 8 – 9 |
| Study risk of bias assessment | 11 | Specify the methods used to assess risk of bias in the included studies, including details of the tool(s) used, how many reviewers assessed each study and whether they worked independently, and if applicable, details of automation tools used in the process. | N/A |
| Effect measures | 12 | Specify for each outcome the effect measure(s) (e.g., risk ratio, mean difference) used in the synthesis or presentation of results. | Table 1 |
| Synthesis methods | 13a | Describe the processes used to decide which studies were eligible for each synthesis (e.g., tabulating the study intervention characteristics and comparing against the planned groups for each synthesis (item #5)). | N/A |
|  | 13b | Describe any methods required to prepare the data for presentation or synthesis, such as handling of missing summary statistics, or data conversions. | N/A |
|  | 13c | Describe any methods used to tabulate or visually display results of individual studies and syntheses. | N/A |
|  | 13d | Describe any methods used to synthesize results and provide a rationale for the choice(s). If meta-analysis was performed, describe the model(s), method(s) to identify the presence and extent of statistical heterogeneity, and software package(s) used. | N/A |
|  | 13e | Describe any methods used to explore possible causes of heterogeneity among study results (e.g., subgroup analysis, meta-regression). | N/A |
|  | 13f | Describe any sensitivity analyses conducted to assess robustness of the synthesized results. | N/A |
| Reporting bias assessment | 14 | Describe any methods used to assess risk of bias due to missing results in a synthesis (arising from reporting biases). | N/A |
| Certainty assessment | 15 | Describe any methods used to assess certainty (or confidence) in the body of evidence for an outcome. | N/A |
| **RESULTS** | | |  |
| Study selection | 16a | Describe the results of the search and selection process, from the number of records identified in the search to the number of studies included in the review, ideally using a flow diagram. | p. 9 |
|  | 16b | Cite studies that might appear to meet the inclusion criteria, but which were excluded, and explain why they were excluded. | Online Supplement 3 |
| Study characteristics | 17 | Cite each included study and present its characteristics. | p. 9 |
| Risk of bias in studies | 18 | Present assessments of risk of bias for each included study. | N/A (Quality assessment in the Online Supplement 5) |
| Results of individual studies | 19 | For all outcomes, present, for each study: (a) summary statistics for each group (where appropriate) and (b) an effect estimate and its precision (e.g., confidence/credible interval), ideally using structured tables or plots. | p. 9 |
| Results of syntheses | 20a | For each synthesis, briefly summarise the characteristics and risk of bias among contributing studies. | N/A |
|  | 20b | Present results of all statistical syntheses conducted. If meta-analysis was done, present for each the summary estimate and its precision (e.g., confidence/credible interval) and measures of statistical heterogeneity. If comparing groups, describe the direction of the effect. | N/A |
|  | 20c | Present results of all investigations of possible causes of heterogeneity among study results. | N/A |
|  | 20d | Present results of all sensitivity analyses conducted to assess the robustness of the synthesized results. | N/A |
| Reporting biases | 21 | Present assessments of risk of bias due to missing results (arising from reporting biases) for each synthesis assessed. | N/A |
| Certainty of evidence | 22 | Present assessments of certainty (or confidence) in the body of evidence for each outcome assessed. | N/A |
| **DISCUSSION** | | |  |
| Discussion | 23a | Provide a general interpretation of the results in the context of other evidence. | p. 13 – 16 |
|  | 23b | Discuss any limitations of the evidence included in the review. | p. 13 – 16 |
|  | 23c | Discuss any limitations of the review processes used. | p. 16 |
|  | 23d | Discuss implications of the results for practice, policy, and future research. | p. 16 – 17 |
| **OTHER INFORMATION** | | |  |
| Registration and protocol | 24a | Provide registration information for the review, including register name and registration number, or state that the review was not registered. | p. 7 |
|  | 24b | Indicate where the review protocol can be accessed, or state that a protocol was not prepared. | N/A |
|  | 24c | Describe and explain any amendments to information provided at registration or in the protocol. | N/A |
| Support | 25 | Describe sources of financial or non-financial support for the review, and the role of the funders or sponsors in the review. | p. 18 |
| Competing interests | 26 | Declare any competing interests of review authors. | p. 18 |
| Availability of data, code and other materials | 27 | Report which of the following are publicly available and where they can be found: template data collection forms; data extracted from included studies; data used for all analyses; analytic code; any other materials used in the review. | N/A  Table 1  Table 1  N/A  Online Supplements |

**Online Supplement 2**

*Applied search terms in the systematic literature search (applied 30^th^ of April 2022)*

(E-Mental Health OR Emental health OR Mobile health OR M-health OR Mhealth OR Emhealth OR Mmhealth OR Digi health OR Digi-health OR Digital health OR Digital mental health OR App-based mental health OR App-based intervention* OR Application based intervention* OR App based intervention* OR Application-based intervention* OR Smartphone delivered intervention* OR Smartphone-delivered intervention* OR Internet delivered OR Internet-based OR internet-intervention* OR internet intervention* OR Internet based OR Internet-based OR Online intervention* OR Internet-delivered OR Internet delivered OR remote OR Smartphone OR online intervention OR online therapy* OR internet-based treatment OR internet based treatment OR web-based treatment OR web based treatment OR web-based intervention* OR webbased intervention* OR technology-delivered* OR technology delivered*) AND (Refugee* OR Asylum seeker* OR Survivor* OR Forced Migration OR Torture OR Victims of Human rights violations OR Flight OR refugee camp*) AND (Mental health OR Depression OR Depress* OR Anxiet* OR Anxiety OR PTSD OR Stress Disorders, Post-Traumatic* OR Mental illness* OR Mental disorder* OR well-being OR quality of life OR Post-migration stress* OR postmigration stress* OR Mental disease* OR Mental problem* OR Substance use OR Substance abuse OR emotional disorder* OR emotional stress OR mood disorder*) NOT (cancer OR stroke)

**Online Supplement 3**

*Inclusion and exclusion of each study report screened after data base search*

| **A** | | **B** | **C** | **D** | **E** |
| --- | --- | --- | --- | --- | --- |
| **Eligibility** 433 publications screened,  424 publications excluded after screening of title, abstract, and full-text | | | | | |
| **Publication/ Article** | | **No smartphone-delivered mental health intervention** | **No refugee population** | **Wrong study design** | **Wrong publication language** |
| Acar | 2019 | x |  |  |  |
| Acierno | 2021 | x |  |  |  |
| Agu | 2022 | x |  |  |  |
| Ahlstrom | 2017 | x |  |  |  |
| Ahmad | 2010 | x |  |  |  |
| Ahumada | 2008 | x |  |  |  |
| Alehagen | 2019 | x |  |  |  |
| Almedom | 2007 | x |  |  |  |
| Alvarez | 2018 | x |  |  |  |
| Amin | 2020 | x |  |  |  |
| Archer | 2015 | x |  |  |  |
| Artime | 2019 | x |  |  |  |
| Artra | 2014 | x |  |  |  |
| Avery | 2019 | x |  |  |  |
| Azoum | 2018 | x |  |  |  |
| Baas | 2020 | x |  |  |  |
| Bademci | 2016 | x |  |  |  |
| Badger | 2011 | x |  |  |  |
| Banducci | 2021 | x |  |  |  |
| Barbee | 2021 | x |  |  |  |
| Barger | 2021 | x |  |  |  |
| Bassilios | 2012 | x |  |  |  |
| Bauer | 2022 | x |  |  |  |
| Ben-Zeev | 2017 | x |  |  |  |
| Ben-Zion | 2018 | x |  |  |  |
| Ben-Zion | 2019 | x |  |  |  |
| Benuto | 2018 | x |  |  |  |
| Bergström | 2015 | x |  |  |  |
| Beuerlein | 2017 | x |  |  |  |
| Biegler | 2016 | x |  |  |  |
| Biernacki | 2014 | x |  |  |  |
| Birnbaum | 2005 | x |  |  |  |
| Bistricky | 2019 | x |  |  |  |
| Bo | 2021 | x |  |  |  |
| Bornefeld-Ettmann | 2018 | x |  |  |  |
| Bortolon | 2018 | x |  |  |  |
| Bourke | 2014 | x |  |  |  |
| Brenner | 2007 | x |  |  |  |
| Breuer | 2017 | x |  |  |  |
| Browne | 2019 | x |  |  |  |
| Bruzsik | 2021 | x |  |  |  |
| Budimir | 2021 | x |  |  |  |
| Buonaugurio | 2015 | x |  |  |  |
| Cabrera | 2021 | x |  |  |  |
| Cacciatore | 2013 | x |  |  |  |
| Cahill | 2021 | x |  |  |  |
| Camuso | 2010 | x |  |  |  |
| Carretta | 2013 | x |  |  |  |
| Carroll | 2017 | x |  |  |  |
| Cassell | 2014 | x |  |  |  |
| Castillo | 2013 | x |  |  |  |
| Charak | 2020 | x |  |  |  |
| Chatty | 2013 | x |  |  |  |
| Chen | 2021 | x |  |  |  |
| Cherestal | 2019 | x |  |  |  |
| Cherestal | 2021 | x |  |  |  |
| Cheung | 2016 | x |  |  |  |
| Chiao | 2005 | x |  |  |  |
| Chittenden | 2011 | x |  |  |  |
| Ciampi | 2009 | x |  |  |  |
| Claudius | 2018 | x |  |  |  |
| Cohen | 2011 | x |  |  |  |
| Constantino | 2007 | x |  |  |  |
| Copeland | 2007 | x |  |  |  |
| Cox | 2018 | x |  |  |  |
| Craig | 2021 | x |  |  |  |
| Crooks | 2020 | x |  |  |  |
| Cuttance | 2017 | x |  |  |  |
| D'Andrea | 2013 | x |  |  |  |
| Dahlgren | 2020 | x |  |  |  |
| Danieli | 2016 | x |  |  |  |
| Davidson | 2017 | x |  |  |  |
| de Carvalho | 2015 | x |  |  |  |
| de Montigny Gauthier | 2019 | x |  |  |  |
| Decker | 2020 | x |  |  |  |
| DeCou | 2019 | x |  |  |  |
| Dixon | 2021 | x |  |  |  |
| Dönmez | 2020 | x |  |  |  |
| Doukas | 2018 | x |  |  |  |
| Drapeau | 2019 | x |  |  |  |
| Driggs | 2018 | x |  |  |  |
| Dupre | 2008 | x |  |  |  |
| Easton | 2014 | x |  |  |  |
| Easton | 2021 | x |  |  |  |
| Eberhardt | 2011 | x |  |  |  |
| Edberg | 2021 | x |  |  |  |
| Ede | 2022 | x |  |  |  |
| Ekblad | 2009 | x |  |  |  |
| El-Khani | 2021 | x |  |  |  |
| Ellis | 2021 | x |  |  |  |
| Ellis | 2020 | x |  |  |  |
| Evans | 2020 | x |  |  |  |
| Every | 2017 | x |  |  |  |
| Ey | 2018 | x |  |  |  |
| Farb | 2001 | x |  |  |  |
| Feigelman | 2020 | x |  |  |  |
| Finn | 2018 | x |  |  |  |
| Fischer | 2011 | x |  |  |  |
| Fletcher | 2019 | x |  |  |  |
| Foley | 2015 | x |  |  |  |
| Formica | 2021 | x |  |  |  |
| Fortier | 2006 | x |  |  |  |
| Fourie | 2011 | x |  |  |  |
| Fowler | 2007 | x |  |  |  |
| Fox | 2020 (b) | x |  |  |  |
| Fox | 2020 (a) | x |  |  |  |
| Fu | 2021 | x |  |  |  |
| Fuchino | 2013 | x |  |  |  |
| Gambino | 2014 | x |  |  |  |
| Garner | 2014 | x |  |  |  |
| Gewirtz-Meydan | 2020 (a) | x |  |  |  |
| Gewirtz-Meydan | 2020 (b) | x |  |  |  |
| Gill | 2012 | x |  |  |  |
| Gilmore | 2020 | x |  |  |  |
| Goel | 2013 | x |  |  |  |
| Goldenberg | 2012 | x |  |  |  |
| Gómez | 2017 | x |  |  |  |
| Gómez | 2015 | x |  |  |  |
| Goodell | 2018 | x |  |  |  |
| Goral | 2020 | x |  |  |  |
| Goverover | 2010 | x |  |  |  |
| Granot | 2011 | x |  |  |  |
| Gray | 2015 | x |  |  |  |
| Green | 2018 | x |  |  |  |
| Green | 2004 | x |  |  |  |
| Griffin | 2020 | x |  |  |  |
| Grinapol | 2019 | x |  |  |  |
| Gruther | 2017 | x |  |  |  |
| Guyon | 2020 | x |  |  |  |
| Haikalis | 2018 | x |  |  |  |
| Hamam | 2021 | x |  |  |  |
| Hamama-Raz | 2017 | x |  |  |  |
| Hamrick | 2019 | x |  |  |  |
| Harnett | 2016 | x |  |  |  |
| Haroz | 2021 | x |  |  |  |
| Harris | 2018 | x |  |  |  |
| Harrison | 2013 | x |  |  |  |
| Harvey | 2009 | x |  |  |  |
| Hassija | 2011 | x |  |  |  |
| Hassija | 2009 | x |  |  |  |
| Heckman | 2017 | x |  |  |  |
| Heringlake | 2013 | x |  |  |  |
| Herres | 2021 | x |  |  |  |
| Holtman | 2011 | x |  |  |  |
| Hopper | 2018 | x |  |  |  |
| Hosmer | 2010 | x |  |  |  |
| Huang | 2019 | x |  |  |  |
| Hurst | 2015 | x |  |  |  |
| Isawi | 2017 | x |  |  |  |
| Iverson | 2019 | x |  |  |  |
| Jackson | 2012 | x |  |  |  |
| Jansen van Rensburg | 2017 | x |  |  |  |
| Janvier | 2012 | x |  |  |  |
| Jefee-Bahloul | 2014 | x |  |  |  |
| Jennissen | 2016 | x |  |  |  |
| Jones | 2008 | x |  |  |  |
| Joscelyne | 2015 | x |  |  |  |
| Karaca Dinç | 2021 | x |  |  |  |
| Kasahara | 2010 | x |  |  |  |
| Katz | 2020 | x |  |  |  |
| Kazlauskas | 2005 | x |  |  |  |
| Kazlauskas | 2012 | x |  |  |  |
| Ke | 2017 | x |  |  |  |
| Kelley | 2019 | x |  |  |  |
| Kelly | 2017 | x |  |  |  |
| Khawaja | 2019 | x |  |  |  |
| Khawaja | 2016 | x |  |  |  |
| Kho | 2016 | x |  |  |  |
| Kirkpatrick | 2017 | x |  |  |  |
| Kirkpatrick | 2015 | x |  |  |  |
| Klein | 2014 | x |  |  |  |
| Kline | 2021 | x |  |  |  |
| Knaevelsrud | 2006 | x |  |  |  |
| Kou | 2017 | x |  |  |  |
| Kowalczewski | 2011 | x |  |  |  |
| Krause | 2004 | x |  |  |  |
| Krysinska | 2014 | x |  |  |  |
| Kuhn | 2016 | x |  |  |  |
| Kurdyla | 2021 | x |  |  |  |
| Labadie | 2018 | x |  |  |  |
| Lahav | 2020 | x |  |  |  |
| Langston | 2019 | x |  |  |  |
| Larsen | 2004 | x |  |  |  |
| Latz | 2020 | x |  |  |  |
| Lazarevic | 2012 | x |  |  |  |
| Leaune | 2021 | x |  |  |  |
| Lee | 2019 | x |  |  |  |
| Lee | 2021 | x |  |  |  |
| Leedom | 2019 | x |  |  |  |
| Leibowitz-Levy | 2005 | x |  |  |  |
| Leone | 2011 | x |  |  |  |
| Lie | 2004 | x |  |  |  |
| Lieppe | 2006 | x |  |  |  |
| Lin | 2020 | x |  |  |  |
| Littleton | 2019 | x |  |  |  |
| Liu | 2018 | x |  |  |  |
| Lizarazo | 2016 | x |  |  |  |
| Lotzin | 2022 | x |  |  |  |
| Lu | 2014 | x |  |  |  |
| Maftoon | 2017 | x |  |  |  |
| Mahamid | 2020 | x |  |  |  |
| Mahoney | 2012 | x |  |  |  |
| Malik | 2017 | x |  |  |  |
| Malisoux | 2022 | x |  |  |  |
| Mao | 2018 | x |  |  |  |
| Maple | 2019 | x |  |  |  |
| Marshall | 2020 | x |  |  |  |
| Marshall | 2005 | x |  |  |  |
| Matthews | 2022 | x |  |  |  |
| McEwen | 2022 | x |  |  |  |
| McGirr | 2018 | x |  |  |  |
| McGirr | 2020 | x |  |  |  |
| McKay | 2017 | x |  |  |  |
| McLean | 2020 | x |  |  |  |
| Meeker | 2021 | x |  |  |  |
| Mello | 2001 | x |  |  |  |
| Melton | 2020 | x |  |  |  |
| Meyer | 2018 | x |  |  |  |
| Miles-McLean | 2022 | x |  |  |  |
| Millegan | 2016 | x |  |  |  |
| Mills | 2012 | x |  |  |  |
| Mittal | 2011 | x |  |  |  |
| Moore | 2019 | x |  |  |  |
| Mori | 2018 | x |  |  |  |
| Mucic | 2008 | x |  |  |  |
| Mucic | 2010 | x |  |  |  |
| Müller | 2021 | x |  |  |  |
| Müllerschön | 2019 | x |  |  |  |
| Munroe | 2022 | x |  |  |  |
| Naeem | 2011 | x |  |  |  |
| Naim | 2014 | x |  |  |  |
| Napreyenko | 2001 | x |  |  |  |
| Nederlander | 2006 | x |  |  |  |
| Nguyen | 2016 | x |  |  |  |
| Niemi | 2017 | x |  |  |  |
| Norup | 2017 | x |  |  |  |
| Oexle | 2020 | x |  |  |  |
| Oganesova | 2022 | x |  |  |  |
| Okumu | 2022 | x |  |  |  |
| Owens-King | 2019 | x |  |  |  |
| Page | 2013 | x |  |  |  |
| Palombo | 2016 | x |  |  |  |
| Pandalangat | 2021 | x |  |  |  |
| Papai | 2020 | x |  |  |  |
| Patel | 2021 | x |  |  |  |
| Payne | 2017 | x |  |  |  |
| Peacock | 2020 | x |  |  |  |
| Pechak | 2020 | x |  |  |  |
| Pegram | 2019 | x |  |  |  |
| Peltonen | 2022 | x |  |  |  |
| Perez | 2009 | x |  |  |  |
| Perron | 2010 | x |  |  |  |
| Persson | 2015 | x |  |  |  |
| Pfefferbaum | 2016 | x |  |  |  |
| Pfeiffer | 2018 | x |  |  |  |
| Pico | 2010 | x |  |  |  |
| Piepzna-Samarasinha | 2011 | x |  |  |  |
| Piotrowicz | 2018 | x |  |  |  |
| Pisani | 2008 | x |  |  |  |
| Pocuca | 2019 | x |  |  |  |
| Port | 2001 | x |  |  |  |
| Posselt | 2019 | x |  |  |  |
| Powell-Dunford | 2014 | x |  |  |  |
| Powell | 2016 | x |  |  |  |
| Quinlan | 2016 | x |  |  |  |
| Racette | 2020 | x |  |  |  |
| Rafful | 2018 | x |  |  |  |
| Raghavan | 2019 | x |  |  |  |
| Ramsay | 2014 | x |  |  |  |
| Rapp | 2005 | x |  |  |  |
| Rellini | 2012 | x |  |  |  |
| Riccio | 2016 | x |  |  |  |
| Riegel | 2009 | x |  |  |  |
| Rietdijk | 2020 | x |  |  |  |
| Rizzo | 2015 | x |  |  |  |
| Roberts | 2021 | x |  |  |  |
| Robertson | 2020 | x |  |  |  |
| Rochefort-Hoehn | 2016 | x |  |  |  |
| Roehrig | 2007 | x |  |  |  |
| Rohr | 2021 | x |  |  |  |
| Rosman | 2017 | x |  |  |  |
| Rucklidge | 2014 | x |  |  |  |
| Rumbold | 2015 | x |  |  |  |
| Ryckman | 2021 | x |  |  |  |
| Saldaña | 2021 | x |  |  |  |
| Saleh | 2018 | x |  |  |  |
| Sandoval | 2017 | x |  |  |  |
| Sanford | 2016 | x |  |  |  |
| Sanhori | 2020 | x |  |  |  |
| Sarno | 2012 | x |  |  |  |
| Sayre | 2004 | x |  |  |  |
| Schiltz | 2014 | x |  |  |  |
| Schlechter | 2021 | x |  |  |  |
| Schlott | 2021 | x |  |  |  |
| Scocco | 2019 | x |  |  |  |
| Sethi | 2013 | x |  |  |  |
| Sevransky | 2021 | x |  |  |  |
| Shafer | 2019 | x |  |  |  |
| Shah | 2019 | x |  |  |  |
| Sirin | 2018 | x |  |  |  |
| Sit | 2019 | x |  |  |  |
| Smith | 2010 | x |  |  |  |
| Smith | 2012 | x |  |  |  |
| Sonderegger | 2011 | x |  |  |  |
| Soral | 2022 | x |  |  |  |
| Spaventa-Vancil | 2016 | x |  |  |  |
| Stanley | 2019 | x |  |  |  |
| Stanley | 2015 | x |  |  |  |
| Steenkamp | 2017 | x |  |  |  |
| Stevens | 2019 | x |  |  |  |
| Strange | 2017 | x |  |  |  |
| Sudore | 2018 | x |  |  |  |
| Syed | 2021 | x |  |  |  |
| Taher | 2020 | x |  |  |  |
| Tang | 2017 | x |  |  |  |
| Tarnanas | 2004 | x |  |  |  |
| Tay | 2018 | x |  |  |  |
| Tay | 2019 (a) | x |  |  |  |
| Tay | 2019 (b) | x |  |  |  |
| Thomsen | 2016 | x |  |  |  |
| Tietjen | 2010 (a) | x |  |  |  |
| Tietjen | 2010 (b) | x |  |  |  |
| Tol | 2007 | x |  |  |  |
| Tomita | 2016 | x |  |  |  |
| Tranter | 2021 | x |  |  |  |
| Trent | 2013 | x |  |  |  |
| Tusa | 2020 | x |  |  |  |
| Tyrer | 2007 | x |  |  |  |
| Upatising | 2015 | x |  |  |  |
| Vaca | 2021 | x |  |  |  |
| Valentine | 2020 | x |  |  |  |
| van der Meer | 2017 | x |  |  |  |
| Vasquez | 2018 | x |  |  |  |
| Vlake | 2022 | x |  |  |  |
| Volgin | 2019 | x |  |  |  |
| Voth Schrag | 2018 | x |  |  |  |
| Voth Schrag | 2020 | x |  |  |  |
| Wachs | 2019 | x |  |  |  |
| Wade | 2014 | x |  |  |  |
| Wagner | 2007 | x |  |  |  |
| Walker | 2015 | x |  |  |  |
| Walsh | 2016 | x |  |  |  |
| Wang | 2020 | x |  |  |  |
| Wang | 2017 | x |  |  |  |
| Wångdahl | 2021 | x |  |  |  |
| Ward-Ciesielski | 2015 | x |  |  |  |
| Webermann | 2020 | x |  |  |  |
| Weingarten | 2021 | x |  |  |  |
| Wéry | 2019 | x |  |  |  |
| Westerlund | 2020 | x |  |  |  |
| Wildman | 2022 | x |  |  |  |
| Wolf | 2015 | x |  |  |  |
| Wu | 2021 | x |  |  |  |
| Wu | 2012 | x |  |  |  |
| Wynaden | 2005 | x |  |  |  |
| Xu | 2011 (a) | x |  |  |  |
| Xu | 2011 (b) | x |  |  |  |
| Xu | 2021 | x |  |  |  |
| Yankah | 2020 | x |  |  |  |
| Young | 2018 | x |  |  |  |
| Yundt | 2020 | x |  |  |  |
| Zheng | 2014 | x |  |  |  |
| Zhou | 2020 | x |  |  |  |
| Zikmund-Fisher | 2007 | x |  |  |  |
| Böttche | 2014 (a) |  | x |  |  |
| Böttche | 2014 (b) |  | x |  |  |
| Böttche | 2016 |  | x |  |  |
| Campos | 2016 |  | x |  |  |
| Campos | 2019 |  | x |  |  |
| Castro | 2019 |  | x |  |  |
| Cipolletta | 2022 |  | x |  |  |
| Constantino | 2015 |  | x |  |  |
| Cox | 2019 |  | x |  |  |
| Cox | 2020 |  | x |  |  |
| Davis | 2021 |  | x |  |  |
| Devane | 2021 |  | x |  |  |
| Dumarkaite | 2021 |  | x |  |  |
| Esfahani | 2016 |  | x |  |  |
| Felsen | 2021 |  | x |  |  |
| Fine | 2018 |  | x |  |  |
| Fiorillo | 2017 |  | x |  |  |
| Fiorillo | 2015 |  | x |  |  |
| Gawlytta | 2017 |  | x |  |  |
| Gawlytta | 2020 |  | x |  |  |
| Geramita | 2020 |  | x |  |  |
| Grenawalt | 2020 |  | x |  |  |
| Gushwa | 2019 |  | x |  |  |
| Heinz | 2021 |  | x |  |  |
| Inmon Long | 2006 |  | x |  |  |
| Knaevelsrud | 2017 |  | x |  |  |
| Knaevelsrud | 2007 |  | x |  |  |
| Knaevelsrud | 2014 |  | x |  |  |
| Knaevelsrud | 2010 (a) |  | x |  |  |
| Knaevelsrud | 2010 (b) |  | x |  |  |
| Kramer | 2015 |  | x |  |  |
| Kuhn | 2017 |  | x |  |  |
| Lerner | 2007 |  | x |  |  |
| Littleton | 2012 |  | x |  |  |
| Littleton | 2016 |  | x |  |  |
| Miner | 2016 |  | x |  |  |
| Mouthaan | 2011 |  | x |  |  |
| Nguyen-Feng | 2016 |  | x |  |  |
| Nguyen-Feng | 2017 |  | x |  |  |
| Nygren | 2019 |  | x |  |  |
| Pantziaras | 2015 |  | x |  |  |
| Quero | 2015 |  | x |  |  |
| Ragavan | 2020 |  | x |  |  |
| Rosenberger | 2016 |  | x |  |  |
| Sabri | 2019 |  | x |  |  |
| Sabri | 2021 |  | x |  |  |
| Steinmetz | 2012 |  | x |  |  |
| Theimer | 2020 |  | x |  |  |
| van Drongelen | 2013 |  | x |  |  |
| van Drongelen | 2014 |  | x |  |  |
| Vechiu | 2021 |  | x |  |  |
| Villegas-Gold | 2018 |  | x |  |  |
| Wagner | 2005 |  | x |  |  |
| Wagner | 2006 |  | x |  |  |
| Wagner | 2007 |  | x |  |  |
| Wagner | 2012 (a) |  | x |  |  |
| Wagner | 2012 (b) |  | x |  |  |
| Wang | 2013 |  | x |  |  |
| Wang | 2016 |  | x |  |  |
| Xu | 2016 |  | x |  |  |
| Böge | 2020 |  |  |  |  |
| Burchert | 2019 |  |  |  |  |
| Fischer | 2021 |  |  |  |  |
| Golchert | 2019 |  |  |  |  |
| Holmes | 2017 |  |  |  |  |
| Lindegaard | 2021 (a) |  |  |  |  |
| Lindegaard | 2021 (b) |  |  |  |  |
| Nickerson | 2020 |  |  |  |  |
| Röhr | 2021 |  |  |  |  |

*Notes.* Red = excluded; green = included; Column A: study report; Column B to E: red = primary reason for exclusion.

**Online Supplement 4**

*Interrater agreement for data extraction*

| **Aim of Intervention** | **Language** | **Type/**  **Length** | **Adaptation** | **Study Reports**  **(Author, Year)** | **Aim of the Study** | **Research Design/ Sample size (% f/m/d)** | **Primary Clinical Outcome/ Efficacy** | **Non-Clinical Outcome** | **App Use/ Drop out** |
| --- | --- | --- | --- | --- | --- | --- | --- | --- | --- |
| 100% | 91.6% | 83.3% | 75.0% | ---- | 100% | 91.6% | 91.6% | 75.0% | 91.6% |

**Online Supplement 5**

*Quality assessment of the included published study reports by study type*

CONSORT 2010 checklist of information to include when reporting a randomised trial (Schulz et al., 2011)

| Section/Topic | Item No | Checklist item | | Nickerson et al.  (2019) | Röhr et al.  (2021) | *P*_0_ |
| --- | --- | --- | --- | --- | --- | --- |
| Title and abstract | | |  | | |  |
|  | 1a | Identification as a randomized trial in the title | | + | + | 100% |
|  | 1b | Structured summary of trial design, methods, results, and conclusions (for specific guidance see CONSORT for abstracts) | | - | - | 100% |
| Introduction | | |  | | |  |
| Background and objectives | 2a | Scientific background and explanation of rationale | | + | + | 100% |
|  | 2b | Specific objectives or hypotheses | | + | + | 100% |
| Methods | | |  | | |  |
| Trial design | 3a | Description of trial design (such as parallel, factorial) including allocation ratio | | - | + | 100% |
|  | 3b | Important changes to methods after trial commencement (such as eligibility criteria), with reasons | | N/A | N/A | 100% |
| Participants | 4a | Eligibility criteria for participants | | + | + | 100% |
|  | 4b | Settings and locations where the data were collected | | - | - | 100% |
| Interventions | 5 | The interventions for each group with sufficient details to allow replication, including how and when they were actually administered | | + | - | 100% |
| Outcomes | 6a | Completely defined pre-specified primary and secondary outcome measures, including how and when they were assessed | | + | + | 100% |
|  | 6b | Any changes to trial outcomes after the trial commenced, with reasons | | N/A | N/A | 100% |
| Sample size | 7a | How sample size was determined | | + | + | 100% |
|  | 7b | When applicable, explanation of any interim analyses and stopping guidelines | | N/A | N/A | 100% |
| Randomisation: |  |  | |  |  |  |
| Sequence generation | 8a | Method used to generate the random allocation sequence | | + | + | 100% |
|  | 8b | Type of randomization; details of any restriction (such as blocking and block size) | | - | + | 100% |
| Allocation concealment mechanism | 9 | Mechanism used to implement the random allocation sequence (such as sequentially numbered containers), describing any steps taken to conceal the sequence until interventions were assigned | | + | + | 50% |
| Implementation | 10 | Who generated the random allocation sequence, who enrolled participants, and who assigned participants to interventions | | + | + | 100% |
| Blinding | 11a | If done, who was blinded after assignment to interventions (for example, participants, care providers, those assessing outcomes) and how | | N/A | + | 100% |
|  | 11b | If relevant, description of the similarity of interventions | | N/A | + | 50% |
| Statistical methods | 12a | Statistical methods used to compare groups for primary and secondary outcomes | | + | + | 100% |
|  | 12b | Methods for additional analyses, such as subgroup analyses and adjusted analyses | | + | + | 100% |
| Results | | |  | | |  |
| Participant flow (a diagram is strongly recommended) | 13a | For each group, the numbers of participants who were randomly assigned, received intended treatment, and were analyzed for the primary outcome | | + | + | 100% |
|  | 13b | For each group, losses and exclusions after randomization, together with reasons | | + | + | 100% |
| Recruitment | 14a | Dates defining the periods of recruitment and follow-up | | - | + | 100% |
|  | 14b | Why the trial ended or was stopped | | - | - | 100% |
| Baseline data | 15 | A table showing baseline demographic and clinical characteristics for each group | | + | + | 100% |
| Numbers analysed | 16 | For each group, number of participants (denominator) included in each analysis and whether the analysis was by original assigned groups | | - | + | 100% |
| Outcomes and estimation | 17a | For each primary and secondary outcome, results for each group, and the estimated effect size and its precision (such as 95% confidence interval) | | + | + | 100% |
|  | 17b | For binary outcomes, presentation of both absolute and relative effect sizes is recommended | | N/A | N/A | 100% |
| Ancillary analyses | 18 | Results of any other analyses performed, including subgroup analyses and adjusted analyses, distinguishing pre-specified from exploratory | | N/A | + | 100% |
| Harms | 19 | All important harms or unintended effects in each group (for specific guidance see CONSORT for harms) | | - | + | 100% |
| Discussion | | |  | | |  |
| Limitations | 20 | Trial limitations, addressing sources of potential bias, imprecision, and, if relevant, multiplicity of analyses | | + | + | 100% |
| Generalisability | 21 | Generalizability (external validity, applicability) of the trial findings | | + | + | 100% |
| Interpretation | 22 | Interpretation consistent with results, balancing benefits and harms, and considering other relevant evidence | | + | + | 100% |
| Other information | | | |  |  |  |
| Registration | 23 | Registration number and name of trial registry | | + | + | 100% |
| Protocol | 24 | Where the full trial protocol can be accessed, if available | | N/A | + | 100% |
| Funding | 25 | Sources of funding and other support (such as supply of drugs), role of funders | | - | + | 50% |

CONSORT 2010 checklist of information to include when reporting a pilot or feasibility trial (Eldridge et al., 2016)

| Section/Topic | Item No | Checklist item | | Holmes et al. (2017) | | Lindegaard et al. (2021b) | Spanhel et al. (2021) | *P*_0_ |
| --- | --- | --- | --- | --- | --- | --- | --- | --- |
| Title and abstract | | |  | |  | | |  |
|  | 1a | Identification as a pilot or feasibility randomised trial in the title | | + | | + | + | 100% |
|  | 1b | Structured summary of pilot trial design, methods, results, and conclusions (for specific guidance see CONSORT abstract extension for pilot trials) | | + | | - | - | 100% |
| Introduction | | |  | |  | | |  |
| Background and objectives | 2a | Scientific background and explanation of rationale for future definitive trial, and reasons for randomised pilot trial | | - | | + | + | 100% |
|  | 2b | Specific objectives or research questions for pilot trial | | + | | + | + | 100% |
| Methods | | |  | |  | | |  |
| Trial design | 3a | Description of pilot trial design (such as parallel, factorial) including allocation ratio | | N/A | | + | + | 100% |
|  | 3b | Important changes to methods after pilot trial commencement (such as eligibility criteria), with reasons | | N/A | | - | + | 100% |
| Participants | 4a | Eligibility criteria for participants | | - | | + | + | 100% |
|  | 4b | Settings and locations where the data were collected | | - | | - | + | 100% |
|  | 4c | How participants were identified and consented | | + | | - | - | 100% |
| Interventions | 5 | The interventions for each group with sufficient details to allow replication, including how and when they were actually administered | | + | | + | - | 100% |
| Outcomes | 6a | Completely defined prespecified assessments or measurements to address each pilot trial objective specified in 2b, including how and when they were assessed | | + | | + | + | 100% |
|  | 6b | Any changes to pilot trial assessments or measurements after the pilot trial commenced, with reasons | | - | | - | N/A | 100% |
|  | 6c | If applicable, prespecified criteria used to judge whether, or how, to proceed with future definitive trial | | + | | - | N/A | 66.67% |
| Sample size | 7a | Rationale for numbers in the pilot trial | | - | | - | - | 66.67% |
|  | 7b | When applicable, explanation of any interim analyses and stopping guidelines | | N/A | | + | N/A | 100% |
| Randomisation: |  |  | |  | |  |  |  |
| Sequence  generation | 8a | Method used to generate the random allocation sequence | | N/A | | + | + | 100% |
|  | 8b | Type of randomisation(s); details of any restriction (such as blocking and block size) | | N/A | | - | + | 100% |
| Allocation  concealment  mechanism | 9 | Mechanism used to implement the random allocation sequence (such as sequentially numbered containers), describing any steps taken to conceal the sequence until interventions were assigned | | N/A | | + | + | 100% |
| Implementation | 10 | Who generated the random allocation sequence, who enrolled participants, and who assigned participants to interventions | | N/A | | + | + | 100% |
| Blinding | 11a | If done, who was blinded after assignment to interventions (for example, participants, care providers, those assessing outcomes) and how | | N/A | | N/A | N/A | 100% |
|  | 11b | If relevant, description of the similarity of interventions | | N/A | | N/A | N/A | 100% |
| Statistical methods | 12 | Methods used to address each pilot trial objective whether qualitative or quantitative | | - | | + | + | 66.67% |
| Results | | |  | |  | | |  |
| Participant flow (a diagram is strongly recommended) | 13a | For each group, the numbers of participants who were approached and/or assessed for eligibility, randomly assigned, received intended treatment, and were assessed for each objective | | - | | + | + | 100% |
|  | 13b | For each group, losses and exclusions after randomisation, together with reasons | | - | | + | - | 100% |
| Recruitment | 14a | Dates defining the periods of recruitment and follow-up | | - | | - | + | 66.67% |
|  | 14b | Why the pilot trial ended or was stopped | | - | | + | - | 66.67% |
| Baseline data | 15 | A table showing baseline demographic and clinical characteristics for each group | | + | | + | + | 100% |
| Numbers analysed | 16 | For each objective, number of participants (denominator) included in each analysis. If relevant, these numbers should be by randomized group | | + | | + | - | 100% |
| Outcomes and estimation | 17 | For each objective, results including expressions of uncertainty (such as 95% confidence interval) for any estimates. If relevant, these results should be by randomized group | | + | | + | + | 100% |
| Ancillary analyses | 18 | Results of any other analyses performed that could be used to inform the future definitive trial | | N/A | | - | + | 66.67% |
| Harms | 19 | All important harms or unintended effects in each group (for specific guidance see CONSORT for harms) | | - | | + | + | 100% |
|  | 19a | If relevant, other important unintended consequences | | N/A | | N/A | N/A | 100% |
| Discussion | | |  | |  | | |  |
| Limitations | 20 | Pilot trial limitations, addressing sources of potential bias and remaining uncertainty about feasibility | | + | | + | + | 100% |
| Generalisability | 21 | Generalizability (applicability) of pilot trial methods and findings to future definitive trial and other studies | | + | | - | + | 100% |
| Interpretation | 22 | Interpretation consistent with pilot trial objectives and findings, balancing potential benefits and harms, and considering other relevant evidence | | + | | + | + | 100% |
|  | 22a | Implications for progression from pilot to future definitive trial, including any proposed amendments | | - | | - | + | 100% |
| Other information | | | |  | |  |  |  |
| Registration | 23 | Registration number for pilot trial and name of trial registry | | - | | + | + | 100% |
| Protocol | 24 | Where the pilot trial protocol can be accessed, if available | | - | | N/A | - | 100% |
| Funding | 25 | Sources of funding and other support (such as supply of drugs), role of funders | | - | | - | - | 100% |
|  | 26 | Ethical approval or approval by research review committee, confirmed with reference number | | + | | + | + | 100% |

**Standards for Reporting Qualitative Research (SRQR; O’Brien et al., 2014)**

| **Section/Topic** | **Items** | **Burchert et al. (2019)** | **Lindegaard et al. (2021a)** | **Lindegaard et al. (2022)** | **Spanhel et al. (2019)** | ***P*_0_** |
| --- | --- | --- | --- | --- | --- | --- |
| Title and Abstract | **Title** - Concise description of the nature and topic of the study Identifying the study as qualitative or indicating the approach (e.g., ethnography, grounded theory) or data collection methods (e.g., interview, focus group) is recommended | - | + | + | - | 75% |
|  | **Abstract** - Summary of key elements of the study using the abstract format of the intended publication; typically includes background, purpose, methods, results, and conclusions | + | + | + | + | 100% |
| Introduction | **Problem formulation** - Description and significance of the problem/phenomenon studied; review of relevant theory and empirical work; problem statement | + | + | + | + | 100% |
|  | **Purpose or research questio**n - Purpose of the study and specific objectives or questions | + | + | + | + | 100% |
| Methods | **Qualitative approach and research paradigm** - Qualitative approach (e.g., ethnography, grounded theory, case study, phenomenology, narrative research) and guiding theory if appropriate; identifying the research paradigm (e.g., postpositivist, constructivist/ interpretivist) is also recommended; rationale** | - | + | - | - | 75% |
|  | **Researcher characteristics and reflexivity** - Researchers’ characteristics that may influence the research, including personal attributes, qualifications/experience, relationship with participants, assumptions, and/or presuppositions; potential or actual interaction between researchers’ characteristics and the research questions, approach, methods, results, and/or transferability | - | - | - | - | 100% |
|  | **Context** - Setting/site and salient contextual factors; rationale** | - | - | - | - | 75% |
|  | **Sampling strategy** - How and why research participants, documents, or events were selected; criteria for deciding when no further sampling was necessary (e.g., sampling saturation); rationale** | + | + | - | + | 100% |
|  | **Ethical issues pertaining to human subjects** - Documentation of approval by an appropriate ethics review board and participant consent, or explanation for lack thereof; other confidentiality and data security issues | + | + | - | + | 100% |
|  | **Data collection methods** - Types of data collected; details of data collection procedures including (as appropriate) start and stop dates of data collection and analysis, iterative process, triangulation of sources/methods, and modification of procedures in response to evolving study findings; rationale** | + | - | - | - | 100% |
|  | **Data collection instruments and technologies** - Description of instruments (e.g., interview guides, questionnaires) and devices (e.g., audio recorders) used for data collection; if/how the instrument(s) changed over the course of the study | + | - | + | + | 100% |
|  | **Units of study** - Number and relevant characteristics of participants, documents, or events included in the study; level of participation (could be reported in results) | + | + | + | + | 100% |
|  | **Data processing** - Methods for processing data prior to and during analysis, including transcription, data entry, data management and security, verification of data integrity, data coding, and anonymization/de-identification of excerpts | - | - | - | + | 100% |
|  | **Data analysis** - Process by which inferences, themes, etc., were identified and developed, including the researchers involved in data analysis; usually references a specific paradigm or approach; rationale** | + | - | - | + | 100% |
|  | **Techniques to enhance trustworthiness** - Techniques to enhance trustworthiness and credibility of data analysis (e.g., member checking, audit trail, triangulation); rationale** | - | - | - | - | 100% |
| Results/findings | **Synthesis and interpretation** - Main findings (e.g., interpretations, inferences, and themes); might include development of a theory or model, or integration with prior research or theory | + | + | + | + | 100% |
|  | **Links to empirical data** - Evidence (e.g., quotes, field notes, text excerpts, photographs) to substantiate analytic findings | + | + | + | + | 100% |
| Discussion | **Integration with prior work, implications, transferability, and contribution(s) to the field -** Short summary of main findings; explanation of how findings and conclusions connect to, support, elaborate on, or challenge conclusions of earlier scholarship; discussion of scope of application/generalizability; identification of unique contribution(s) to scholarship in a discipline or field | + | + | + | + | 100% |
|  | **Limitations** - Trustworthiness and limitations of findings | + | + | + | + | 100% |
| Other | **Conflicts of interest** - Potential sources of influence or perceived influence on study conduct and conclusions; how these were managed | + | + | + | + | 100% |
|  | **Funding** - Sources of funding and other support; role of funders in data collection, interpretation, and reporting | - | + | - | + | 100% |

**The rationale should briefly discuss the justification for choosing that theory, approach, method, or technique rather than other options available, the assumptions and limitations implicit in those choices, and how those choices influence study conclusions and transferability. As appropriate, the rationale for several items might be discussed together.

**SPIRIT 2013 Checklist: Recommended items to address in a clinical trial protocol and related documents (Chan et al., 2013)**

| Section/item | ItemNo | Description | Böge et al. (2020) | Fischer et al. (2021) | *P*_0_ |  |
| --- | --- | --- | --- | --- | --- | --- |
| **Administrative information** | | |  |  |  |  |
| Title | 1 | Descriptive title identifying the study design, population, interventions, and, if applicable, trial acronym | + | + | 100% |  |
| Trial registration | 2a | Trial identifier and registry name. If not yet registered, name of intended registry | + | + | 100% |  |
|  | 2b | All items from the World Health Organization Trial Registration Data Set | - | - | 100% |  |
| Protocol version | 3 | Date and version identifier | N/A | N/A | 100% |  |
| Funding | 4 | Sources and types of financial, material, and other support | + | + | 100% |  |
| Roles and responsibilities | 5a | Names, affiliations, and roles of protocol contributors | - | + | 100% |  |
|  | 5b | Name and contact information for the trial sponsor | - | - | 100% |  |
|  | 5c | Role of study sponsor and funders, if any, in study design; collection, management, analysis, and interpretation of data; writing of the report; and the decision to submit the report for publication, including whether they will have ultimate authority over any of these activities | - | - | 100% |  |
|  | 5d | Composition, roles, and responsibilities of the coordinating center, steering committee, endpoint adjudication committee, data management team, and other individuals or groups overseeing the trial, if applicable (see Item 21a for data monitoring committee) | - | - | 100% |  |
| Introduction |  |  |  |  |  |  |
| Background and rationale | 6a | Description of research question and justification for undertaking the trial, including summary of relevant studies (published and unpublished) examining benefits and harms for each intervention | - | + | 66.67% |  |
|  | 6b | Explanation for choice of comparators | - | - | 66.67% |  |
| Objectives | 7 | Specific objectives or hypotheses | + | + | 100% |  |
| Trial design | 8 | Description of trial design including type of trial (e.g., parallel group, crossover, factorial, single group), allocation ratio, and framework (e.g., superiority, equivalence, noninferiority, exploratory) | + | + | 100% |  |
| Methods: Participants, interventions, and outcomes | | |  |  |  |  |
| Study setting | 9 | Description of study settings (e.g., community clinic, academic hospital) and list of countries where data will be collected. Reference to where list of study sites can be obtained | + | + | 66.67% |  |
| Eligibility criteria | 10 | Inclusion and exclusion criteria for participants. If applicable, eligibility criteria for study centres and individuals who will perform the interventions (e.g., surgeons, psychotherapists) | + | + | 100% |  |
| Interventions | 11a | Interventions for each group with sufficient detail to allow replication, including how and when they will be administered | + | + | 100% |  |
|  | 11b | Criteria for discontinuing or modifying allocated interventions for a given trial participant (e.g., drug dose change in response to harms, participant request, or improving/worsening disease) | + | + | 100% |  |
|  | 11c | Strategies to improve adherence to intervention protocols, and any procedures for monitoring adherence (e.g., drug tablet return, laboratory tests) | + | + | 100% |  |
|  | 11d | Relevant concomitant care and interventions that are permitted or prohibited during the trial | - | + | 66.67% |  |
| Outcomes | 12 | Primary, secondary, and other outcomes, including the specific measurement variable (e.g., systolic blood pressure), analysis metric (e.g., change from baseline, final value, time to event), method of aggregation (e.g., median, proportion), and time point for each outcome. Explanation of the clinical relevance of chosen efficacy and harm outcomes is strongly recommended | + | + | 100% |  |
| Participant timeline | 13 | Time schedule of enrolment, interventions (including any run-ins and washouts), assessments, and visits for participants. A schematic diagram is highly recommended (see Figure) | + | + | 100% |  |
| Sample size | 14 | Estimated number of participants needed to achieve study objectives and how it was determined, including clinical and statistical assumptions supporting any sample size calculations | + | - | 100% |  |
| Recruitment | 15 | Strategies for achieving adequate participant enrolment to reach target sample size | - | + | 100% |  |
| **Methods: Assignment of interventions (for controlled trials)** | | |  |  |  |  |
| Allocation: |  |  |  |  |  |  |
| Sequence generation | 16a | Method of generating the allocation sequence (e.g., computer-generated random numbers), and list of any factors for stratification. To reduce predictability of a random sequence, details of any planned restriction (e.g., blocking) should be provided in a separate document that is unavailable to those who enroll participants or assign interventions | + | N/A | 100% |  |
| Allocation concealment mechanism | 16b | Mechanism of implementing the allocation sequence (e.g., central telephone; sequentially numbered, opaque, sealed envelopes), describing any steps to conceal the sequence until interventions are assigned | + | N/A | 100% |  |
| Implementation | 16c | Who will generate the allocation sequence, who will enroll participants, and who will assign participants to interventions | + | N/A | 100% |  |
| Blinding (masking) | 17a | Who will be blinded after assignment to interventions (e.g., trial participants, care providers, outcome assessors, data analysts), and how | + | N/A | 100% |  |
|  | 17b | If blinded, circumstances under which unblinding is permissible, and procedure for revealing a participant’s allocated intervention during the trial | - | N/A | 100% |  |
| **Methods: Data collection, management, and analysis** | | |  |  |  |  |
| Data collection methods | 18a | Plans for assessment and collection of outcome, baseline, and other trial data, including any related processes to promote data quality (e.g., duplicate measurements, training of assessors) and a description of study instruments (e.g., questionnaires, laboratory tests) along with their reliability and validity, if known. Reference to where data collection forms can be found, if not in the protocol | + | - | 100% |  |
|  | 18b | Plans to promote participant retention and complete follow-up, including list of any outcome data to be collected for participants who discontinue or deviate from intervention protocols | - | + | 100% |  |
| Data management | 19 | Plans for data entry, coding, security, and storage, including any related processes to promote data quality (e.g., double data entry; range checks for data values). Reference to where details of data management procedures can be found, if not in the protocol | + | + | 66.67% |  |
| Statistical methods | 20a | Statistical methods for analyzing primary and secondary outcomes. Reference to where other details of the statistical analysis plan can be found, if not in the protocol | + | + | 100% |  |
|  | 20b | Methods for any additional analyses (e.g., subgroup and adjusted analyses) | + | - | 100% |  |
|  | 20c | Definition of analysis population relating to protocol non-adherence (e.g., as randomized analysis), and any statistical methods to handle missing data (e.g., multiple imputation) | + | - | 100% |  |
| **Methods: Monitoring** | | |  |  |  |  |
| Data monitoring | 21a | Composition of data monitoring committee (DMC); summary of its role and reporting structure; statement of whether it is independent from the sponsor and competing interests; and reference to where further details about its charter can be found, if not in the protocol. Alternatively, an explanation of why a DMC is not needed | - | - | 50% |  |
|  | 21b | Description of any interim analyses and stopping guidelines, including who will have access to these interim results and make the final decision to terminate the trial | - | - | 100% |  |
| Harms | 22 | Plans for collecting, assessing, reporting, and managing solicited and spontaneously reported adverse events and other unintended effects of trial interventions or trial conduct | - | - | 66.67% |  |
| Auditing | 23 | Frequency and procedures for auditing trial conduct, if any, and whether the process will be independent from investigators and the sponsor | - | - | 100% |  |
| Ethics and dissemination | | |  |  |  |  |
| Research ethics approval | 24 | Plans for seeking research ethics committee/institutional review board (REC/IRB) approval | + | + | 100% |  |
| Protocol amendments | 25 | Plans for communicating important protocol modifications (e.g., changes to eligibility criteria, outcomes, analyses) to relevant parties (e.g., investigators, REC/IRBs, trial participants, trial registries, journals, regulators) | - | - | 100% |  |
| Consent or assent | 26a | Who will obtain informed consent or assent from potential trial participants or authorized surrogates, and how (see Item 32) | - | + | 100% |  |
|  | 26b | Additional consent provisions for collection and use of participant data and biological specimens in ancillary studies, if applicable | N/A | N/A | 100% |  |
| Confidentiality | 27 | How personal information about potential and enrolled participants will be collected, shared, and maintained in order to protect confidentiality before, during, and after the trial | + | - | 100% |  |
| Declaration of interests | 28 | Financial and other competing interests for principal investigators for the overall trial and each study site | - | + | 100% |  |
| Access to data | 29 | Statement of who will have access to the final trial dataset, and disclosure of contractual agreements that limit such access for investigators | + | - | 100% |  |
| Ancillary and post-trial care | 30 | Provisions, if any, for ancillary and post-trial care, and for compensation to those who suffer harm from trial participation | - | - | 50% |  |
| Dissemination policy | 31a | Plans for investigators and sponsor to communicate trial results to participants, healthcare professionals, the public, and other relevant groups (e.g., via publication, reporting in results databases, or other data sharing arrangements), including any publication restrictions | - | - | 100% |  |
|  | 31b | Authorship eligibility guidelines and any intended use of professional writers | - | - | 100% |  |
|  | 31c | Plans, if any, for granting public access to the full protocol, participant-level dataset, and statistical code | - | - | 100% |  |
| Appendices |  |  |  |  |  |  |
| Informed consent materials | 32 | Model consent form and other related documentation given to participants and authorized surrogates | - | - | 100% |  |
| Biological specimens | 33 | Plans for collection, laboratory evaluation, and storage of biological specimens for genetic or molecular analysis in the current trial and for future use in ancillary studies, if applicable | N/A | N/A | 100% |  |

*Notes:* +/green = reported, -/red = not reported, N/A/orange = Not applicable; *P*_0_ = Observed interrater agreement.
